# Use of benzodiazepine and related drugs in migrants and Finnish-born persons: a nationwide register-based study

**DOI:** 10.1177/14034948221112470

**Published:** 2022-07-25

**Authors:** Venla Lehti, Essi Salama, Solja Niemelä, Antti Tanskanen, Mika Gissler, Jaana Suvisaari, Heidi Taipale

**Affiliations:** 1 Finnish Institute for Health and Welfare, Equality Unit, Finland; 2 University of Helsinki and Helsinki University Hospital; Department of Psychiatry, Finland; 3 Doctoral Programme for Clinical Investigation, Faculty of Medicine, University of Turku, Finland; 4 Department of Child Psychiatry, Turku University Hospital, Finland; 5 Department of Psychiatry, University of Turku, Finland; 6 Department of Psychiatry, Addiction Psychiatry Unit, Turku University Hospital, Finland; 7 University of Eastern Finland, Department of Forensic Psychiatry, Niuvanniemi Hospital, Kuopio, Finland; 8 Karolinska Institutet, Department of Clinical Neuroscience, Stockholm, Sweden; 9 Finnish Institute for Health and Welfare, Impact Assessment Unit, Finland; 10 Karolinska Institutet, Department of Molecular Medicine and Surgery and Region Stockholm, Academic Primary Health Care Centre, Sweden; 11 Finnish Institute for Health and Welfare, Department of Knowledge Brokers, Helsinki, Finland; 12 University of Turku, Research Centre for Child Psychiatry, Finland; 13 University of Eastern Finland, School of Pharmacy, Kuopio, Finland

**Keywords:** Anxiolytics, benzodiazepines, migrants

## Abstract

**Aims:**

Benzodiazepines and related drugs (BZDR) are often used longer than generally recommended. The aim is to study patterns of use among migrant and Finnish-born users of BZDR, and to identify factors that are associated with long-term use and BZDR polytherapy.

**Methods:**

This register-based study includes a nationwide sample of migrants (*n*=8729) and their Finnish-born controls (*n*=11 388) who had purchased BZDR in 2011–2014, but not in 2009–2010. Information on drug purchases was obtained from the National Prescription Register and the duration of drug use was estimated using PRE2DUP method. The main outcomes were long-term use of BZDR, polytherapy and time until discontinuation of BZDR use. Sociodemographic variables and information on preceding psychiatric diagnoses were included as covariates. Logistic and Cox regression analyses were the statistical methods used.

**Results:**

Only migrants from Sub-Saharan Africa were more likely to discontinue the medication once initiated than Finnish-born users. Migrants were significantly less likely to be long-term users (adjusted odds ratio 0.79, 95% CI 0.70–0.89) or polytherapy users (aOR 0.90, 95% CI 0.84–0.97) of BZDR compared with Finnish-born participants.

**Conclusions:**

Migrants had less long-term and concomitant use of several BZDR than Finnish-born participants. The pattern of use is more optimal among migrants, but it may also reflect poorer access to mental health treatment.

## Introduction

Many migrant populations, especially refugees, have a high burden of psychological distress. Traumatic events, migration and daily stressors in the host country expose migrants to mental health problems [1]. Several studies have shown a higher incidence of psychiatric disorders among migrants than non-migrants [1,2]. In Finland, a population-based survey showed higher levels of symptoms of depression and anxiety among migrants compared with non-migrants. [3]. However, the incidence of diagnosed psychiatric disorders is generally lower among migrants than Finnish-born people, except for post-traumatic stress disorder (PTSD), which is more common among migrants [4]. Migrants use fewer psychiatric services and receive less intensive care than Finnish-born people [5]. The most common reason for migration to Finland is employment [6]. In 2021, only 5% of those who were granted a residence permit got it on the basis of international protection [6].

Studying patterns of use of psychotropic medication in different groups of migrants may help in identifying gaps in their psychiatric treatment and possible underuse, as well as overuse or misuse of medication. Benzodiazepines are generally well tolerated and effective for anxiety disorders [7,8]. However, long-term use may be associated with adverse effects and some users develop dependency or misuse these drugs [9,10]. Similarly, non-benzodiazepine hypnotics (Z-drugs) may improve sleep outcomes in short-term use, but are associated with adverse effects, especially among older adults [11]. Even though not generally recommended, a large proportion of incident users of benzodiazepines and related drugs (BZDR) become long-term users [12]. Some users try to cope with psychological distress or discomfort associated with somatic disorder, while others aim at alleviating symptoms of substance-related disorders, or use BZDR for recreational purposes [13]. People with psychiatric disorders, substance use and low socioeconomic status are more likely to misuse benzodiazepines [14]. Deliberate abuse of benzodiazepines is more common among those who use them in conjunction with other substances and obtain the drugs from illicit sources, or who prefer drugs with rapid onset of effect [10,14].

The knowledge on the use of BZDR among migrants is mostly based on register-based studies conducted in Europe. Fewer purchases of anxiolytics were reported in four different groups of migrants in both Spain and Norway when compared with non-migrants [15]. In Northern Ireland, fewer anxiolytics and hypnotics were purchased among migrants than non-migrants, except for those of German origin who did not differ from non-migrants [16]. In the Netherlands, migrants of Turkish, Moroccan and Surinamese origin were shown to have an increased incidence of anxiolytic purchases, but those from the Netherlands Antilles did not when compared with the Dutch population [17]. In Norway, the purchases of anxiolytics and hypnotics or sedatives were less common among most groups of immigrant women compared with Norwegian women [18], but refugee women were more likely to purchase hypnotics than other migrant women [19]. In Sweden, the use of anxiolytics and hypnotics was more common among refugees with longer duration of residence compared with recently migrated refugees [20]. In addition, an Israeli survey showed more use of anxiolytics and hypnotics among migrants, most of whom originated from the former Soviet Union, than among non-migrants [21]. A Spanish survey showed similar patterns of non-medical use of benzodiazepines among migrant and non-migrant adolescents [22]. Several studies have also been published related to ethnic minorities in the United States, where most studies show that misuse of benzodiazepines is more common among non-Hispanic whites than among other ethnic groups [14].

Previous studies on the use of BZDR among migrants have some limitations. Most studies included migrants from few selected countries [15,17–20] or all migrants pooled as one group [21,22]. The use of BZDR was mostly studied as a dichotomous variable [15–22]. Furthermore, the duration of use was not included in any of the previous studies. In some studies, drug use was based on a self-report [21,22]. The use of BZDR was not usually studied in relation to psychiatric disorders [15–22].

In Finland, migrants generally purchase fewer psychotropic drugs than non-migrants [23], and they are less likely to purchase anxiolytics and hypnotics or sedatives than Finnish-born people, regardless of their region of origin or duration of residence [23]. In this study, we focus on migrant and Finnish-born users of BZDR in Finland. The aim is to study patterns of use among incident users, and to identify factors that are associated with long-term use and BZDR polytherapy.

## Methods

The study is a retrospective, register-based study that covers the whole adult migrant population in Finland and a Finnish-born comparison cohort. The study protocol was approved by the Ethics Committee of the Finnish Institute for Health and Welfare (THL) in Finland (589/2013 and 798/2018). The data-keeping organisations authorised the use of register data.

### Participants

The participants in the original sample were identified from the Population Information System (PIS), a national register that contains information about Finnish citizens and permanent residents in Finland. Migrants were born abroad and their mother tongue was not Finnish. Participants had to be at least 15 years old, alive, and resident in Finland on 31 December 2010. To collect a control group, one Finnish-born person matched by age, sex and place of residence was originally identified from the PIS for each migrant. The number of both migrants and their Finnish-born controls in the original sample was 185 605. The participants were followed until 31 December 2015, date of death or emigration from Finland. A more detailed description of the sample has been reported earlier (24).

For this study, only those participants were included who had used BZDR in 2011–2014 and had not used them in 2009–2010. These criteria were chosen to include incident users and to have at least one year of follow-up since initiation. The number of participants who were included was 20,117 (8729 migrants and 11,388 Finnish-born participants).

### Information on drug purchases and periods of drug use

All permanent residents in Finland are covered by the National Health Insurance and all purchases of reimbursed prescription drugs are recorded in the National Prescription Register, which is maintained by KELA, the Social Insurance Institution of Finland. The use of drugs administered during hospital stays is not included. The register covers information on the patient, prescriber and the prescribed drug. In this study we included the purchases of benzodiazepines (Anatomic Therapeutic Chemical classes N05BA, except for anti-epileptic drugs clobazam N05BA09 and clonazepam N03AE01) and drugs related to benzodiazepines (Z-drugs, ATC class N05CF) in 2011–2015. However, the reimbursement of temazepam, nitrazepam and chlordiazepoxide ended before 2015 and were therefore not included in the register until the end of the follow-up period. They were excluded from the analyses to avoid misclassification of discontinuations when these administrative decisions to withdraw reimbursement took place during our study period. Midazolam could not be included either because its reimbursement had already ended before the study started.

The duration of drug use periods were estimated using PRE2DUP method [25]. Sliding averages of defined daily doses were calculated according to individual drug use patterns. Hospital care periods, stockpiling of drugs, variation in purchase events and changing dose were considered. Duration of ‘any BZDR’ use was calculated by combining overlapping periods of all included drugs.

When the outcome was the time to discontinuation of the first period of BZDR use, the period started from the initiation of the first BZDR at the beginning of 2011 and ended when the use of the drug was stopped, the participant died, emigrated from Finland or was hospitalised for more than 90 days, whichever came first.

When studying the patterns of use, the focus was on long-term use and polytherapy. To ensure equal follow-up time, the patterns were studied during the first year since initiation of first BZDR. Long-term use was defined as at least 180 days of continuous use of BZDR. Polytherapy was defined as having at least one overlapping period of at least two different drugs. There was no minimum length for the overlapping period. All participants were included in the main analyses, but additional analyses were conducted without those who had died (*n*=108), emigrated from Finland (*n*=9) or been hospitalised for more than 90 days (*n*=4) during the first year since initiation of drug use.

### Migration-related variables

Migrants were classified by their region of origin and the time they had lived in Finland. Those whose country of origin was unknown and could not be classified based on their language, and those who came from regions with very few participants, were not included in the regional analyses. The regional categories included: (a) Finland; (b) EU/European Free Trade Association (EFTA), North America and Australia; (c) Russia, the former Soviet Union and Eastern Europe (former Eastern European countries not members of the EU); (d) North Africa and the Middle East; (e) Sub-Saharan Africa; and (f) Asia. Time since migration included the following categories: (a) Finnish-born; (b) migrant, moved to Finland less than five years before study start; (c) migrant, moved to Finland 5–15 years before study start; and (d) migrant, moved to Finland more than 15 years before study start.

### Variables related to sociodemographic background

Sociodemographic factors included in the study were sex, age, marital status, socioeconomic status (SES) and receiving social assistance. Information on sex, age and marital status was retrieved from the PIS. Age at the beginning of the study was categorised in four categories: 15–29, 30–44, 45–59 and 60 years or more. There were two categories for marital status: married or in a registered partnership, and unmarried (including those who were widowed, separated or divorced). Information on SES was provided by Statistics Finland. It was primarily based on occupation in 2010 [26]. A five-categorical variable was used: (a) entrepreneurs; (b) upper white-collar workers; (c) lower white-collar workers; (d) blue-collar workers; and (e) others (people not in employment). Information on receiving social assistance, which is financial support for those whose income is insufficient to cover their daily essential expenses, was collected from the Register of Social Assistance maintained by THL. The data was available for years 2009–2011. It was categorised into two categories: (a) the participant or his/her spouse received assistance for at least one month; or (b) did not receive assistance during this period.

### Psychiatric morbidity

Information on psychiatric diagnoses preceding the use of benzodiazepines was retrieved from the Care Register for Health Care (CRHC). CRHC is a nationwide register maintained by THL. In addition to diagnoses, it covers the days of admission and discharge in all inpatient care units and visits in all public specialised outpatient care units in Finland. Information was collected on all psychiatric diagnoses the participants had received during the three years before initiating first benzodiazepine. Diagnostic categories or specific diagnoses included: (a) alcohol-related disorders (code F10 in International Statistical Classification of Diseases and Related Health Problems, 10^th^ revision); (b) other substance use disorders (F11-F16 and F18-F19); (c); psychotic disorders (F20-29); (d) depression (F32-33); (e); bipolar disorder (F30-31); (f) anxiety disorders (F40-42); (g) post-traumatic stress disorder (PTSD, code F43.1); and (h) personality disorders (F60). However, for analyses stratified by migration status, bipolar disorder was excluded, and PTSD was combined with anxiety disorders because of small number of participants (less than ten among either migrants or Finnish-born participants).

### Statistical analysis

The characteristics of migrant and Finnish-born participants were compared using a chi-square test. The duration of BZDR use was compared between migrants and Finnish-born controls using a *t*-test. The time to discontinuation of first period of benzodiazepine use was studied using Cox regression analysis. Logistic regression analysis was used for studying the likelihood of using long-term treatment (versus only short-term use) and polytherapy (versus only one drug at a time) among migrants compared with Finnish-born participants. Both unadjusted odds ratios and those adjusted for sociodemographic variables and preceding diagnoses are reported. When analysing factors which were associated with long-term treatment and polytherapy, logistic regression analyses were stratified by migrant status. A *p* value less than 0.05 was considered statistically significant in all analyses. All analyses were conducted by SPSS version 27.

## Results

### Characteristics of users

The characteristics of migrant and Finnish-born users of BZDRs are shown in Table I. A higher proportion of migrants were female, married and receivers of social assistance when compared with Finnish-born participants. The distribution of age and SES categories also differed significantly between these two groups. Most psychiatric disorders were less common among migrants. PTSD was more common among migrants and no difference was found in the diagnoses of psychotic disorder or depression.

**Table I.** Characteristics of participants who had used benzodiazepines during follow-up.

	Migrants (total *n*=8729), % (*n*)	Finnish-born (*n*=11 388), % (*n*)	*p*
Sex			
Female	59.1 (5155)	56.4 (6420)	<0.001
Age			<0.001
15–29	21.6 (1849)	26.8 (3007)	
30–44	39.8 (3398)	42.7 (4796)	
45–59	29.8 (2549)	24.5 (2752)	
⩾60	8.7 (746)	6.1 (684)	
Socioeconomic status			<0.001
Entrepreneurs	6.2 (503)	4.8 (531)	
Upper white-collar	12.0 (968)	20.4 (2238)	
Lower white-collar	17.3 (1400)	32.0 (3515)	
Blue collar	27.1 (2191)	18.4 (2019)	
Others	37.3 (3014)	24.4 (2679)	
Received social assistance	32.2 (2812)	15.3 (1740)	<0.001
Marital status			<0.001
Married/registered partnership	55.2 (4406)	38.4 (4373)	
Others	44.8 (3572)	61.6 (7015)	
Psychiatric diagnoses (preceding 3 years)			
Alcohol-related disorder (F10)	1.0 (85)	2.5 (281)	<0.001
Other substance use disorder (F11)	0.6 (54)	1.1 (119)	0.001
Psychotic disorder (F20)	2.3 (197)	2.4 (273)	0.540
Depression (F32–33)	5.5 (476)	5.8 (666)	0.243
Bipolar disorder (F30–31)	0.7 (57)	1.2 (142)	<0.001
Anxiety disorder (F40–42)	2.7 (238)	4.0 (455)	<0.001
Post-traumatic stress disorder (F43.1)	0.8 (67)	0.3 (30)	<0.001
Personality disorder (F60)	0.5 (47)	1.3 (148)	<0.001
Any psychiatric disorder (F00–99)	11.5 (1003)	13.7 (1559)	<0.001
Region of origin			
EU/EFTA, North America or Australia	33.5 (2850)	-	
Russia and Eastern Europe	36.2 (3080)	-	
North Africa and the Middle East	14.1 (1202)	-	
Sub-Saharan Africa	6.3 (538)	-	
Asia	9.9 (839)	-	
Time since migration			
Less than 5 years	30.1 (2437)	-	
5–15 years	39.2 (3173)	-	
More than 15 years	30.7 (2487)	-	

### Duration of use

The mean duration of the first period of a BZDR did not differ between migrants and Finnish-born participants. It was 72.5 days for migrants and 70.7 days for Finnish-born participants (*p*=0.451).

Cox regression analysis was used to study the time to discontinuation of the first BZDR. The results are shown in Table II. In unadjusted analyses, most migrant groups showed longer duration of use. When adjusted for sex, age, SES, social assistance, marital status and six most common categories of preceding diagnoses (alcohol use disorder, other substance use disorder, psychosis, depression, anxiety and PTSD and personality disorder), earlier discontinuation of medication was found among migrants from Sub-Saharan Africa (aHR 1.20, 95% CI 1.08–1.33) when compared with the Finnish-born participants.

**Table II.** Cox regression analysis, time to discontinuation of the first benzodiazepine, unadjusted and adjusted hazard ratios.

	HR (95% CI)	aHR (95% CI)
**Migrant status (cf. Finnish-born)**		
Migrant	**0.95 (0.92–0.98)**	1.01 (0.98–1.05)
**Region of origin (cf. Finland)**		
EU/EFTA, North America or Australia	**0.94 (0.90–0.98)**	0.99 (0.94–1.03)
Russia and Eastern Europe	**0.91 (0.87–0.95)**	0.99 (0.94–1.03)
North Africa and the Middle East	**0.93 (0.88–0.99)**	1.06 (0.99–1.14)
Sub-Saharan Africa	**1.11 (1.02–1.21)**	**1.20 (1.08–1.33)**
Asia	1.05 (0.98–1.13)	1.07 (0.99–1.15)
**Time since migration (cf. Finnish-born)**		
Less than 5 years	0.99 (0.95–1.04)	1.03 (0.97–1.08)
5-15 years	**0.96 (0.92–0.999)**	1.02 (0.98–1.07)
More than 15 years	**0.94 (0.90–0.98)**	1.02 (0.97–1.07)

aHR: adjusted for sex, age, SES, social assistance, marital status, alcohol use disorder, other substance use disorder, psychosis, depression, anxiety (including PTSD) and personality disorder. cf: compared against.

### Specific drugs and patterns of use

The specific drugs, which the participants used during the follow-up and the first drug they used, are reported in the Supplementary Table I. The most common drugs used were oxazepam, zopiclone and zolpidem among both migrants and Finnish-born participants. The first drug was more often zopiclone among migrant than Finnish-born users, and it was more often oxazepam, diazepam, zolpidem or several concomitant drugs among Finnish-born than migrant users. The same pattern was found when all drugs used during the follow-up were included.

Patterns of use were studied during the first year since initiation of the first drug. The proportion of those who became long-term users (at least 180 days of continuous use) or polytherapy users (at least two drugs simultaneously) are shown in Table III. It also includes the results of logistic regression analyses.

**Table III.** Risk of long-term use and polytherapy by migration status during the first year. Unadjusted and adjusted binary logistic regression analyses.

	Long-term use (⩾180 days)			Polytherapy		
	% (*n*)	OR (95 % CI)	aOR (95% CI)	% (*n*)	OR (95 % CI)	aOR (95% CI)
**Migrant status**						
Finnish-born (ref)	9.4 (1066)			27.7 (3149)		
Migrant	8.9 (773)	0.94 (0.85–1.04)	**0.79 (0.70–0.89)**	24.9 (2175)	**0.87 (0.82–0.93)**	**0.90 (0.84–0.97)**
**Region of origin**						
Finland (ref)	9.4 (1066)			27.7 (3149)		
EU/EFTA, North America or Australia	9.5 (271)	1.02 (0.88–1.17)	0.94 (0.79–1.11)	26.6 (759)	0.95 (0.87–1.04)	0.99 (0.89–1.10)
Russia and Eastern Europe	9.5 (292)	1.01 (0.89–1.16)	**0.82 (0.69–0.96)**	25.6 (789)	**0.90 (0.82–0.99)**	0.94 (0.85–1.04)
North Africa and the Middle East	9.2 (110)	0.98 (0.79–1.20)	**0.57 (0.44–0.74)**	21.7 (261)	**0.73 (0.63–0.84)**	**0.69 (0.58–0.81)**
Sub-Saharan Africa	5.4 (29)	**0.55 (0.38–0.81)**	**0.42 (0.27–0.66)**	23.0 (124)	**0.78 (0.64–0.96)**	0.81 (0.64–1.03)
Asia	6.3 (53)	**0.65 (0.49–0.87)**	**0.67 (0.49–0.92)**	21.0 (176)	**0.70 (0.59–0.82)**	**0.74 (0.62–0.89)**
**Time since migration**						
Finnish-born (ref)	9.4 (1066)			27.7 (3149)		
Less than 5 years	8.0 (196)	**0.85 (0.72–0.99)**	**0.75 (0.61–0.93)**	22.7 (553)	1.08 (0.98–1.19)	**0.81 (0.71–0.92)**
5–15 years	8.4 (267)	0.89 (0.77–1.02)	**0.79 (0.68–0.93)**	25.1 (798)	**0.83 (0.73–0.94)**	0.93 (0.84–1.02)
More than 15 years	9.5 (236)	1.02 (0.88–1.18)	0.86 (0.73–1.01)	26.2 (652)	0.95 (0.84–1.07)	0.95 (0.85–1.05)

aOR: adjusted for sex, age, SES, social assistance, marital status, alcohol use disorder, other substance use disorder, psychosis, depression, anxiety (including PTSD) and personality disorder.

Migrants were significantly less likely to be long-term users when adjusted for sex, age, SES, social assistance, marital status and preceding diagnoses (aOR 0.76, 95% CI 0.68–0.85). Of the regions of origin, only migrants from EU/EFTA, North America or Australia did not differ from Finnish-born participants. All others were less likely to become long-term users. Migrants from Sub-Saharan Africa had particularly low likelihood of long-term use (aOR 0.42, 95% CI 0.27–0.66). Migrants who had migrated less than five or 5–15 years before the study start were less likely to become long-term users than Finnish-born participants.

Migrants were also less likely to become polytherapy users (aOR 0.90, 95% CI 0.84–0.97). The regional analysis showed that migrants from North Africa and the Middle East and Asia had a lower risk of polytherapy, while others did not differ from Finnish-born participants. Those who had migrated less than five years previously were less likely to use several drugs when compared with Finnish-born users.

The logistic regression analyses were also conducted without participants who had died, emigrated from Finland or been hospitalised for more than 90 days during the first year since initiation of BZDR use. The results are shown in the Supplementary Table II. They were very similar to the results described above.

### Characteristics associated with long-term use and polytherapy

To identify possible differences in the factors that are associated with long-term use or polytherapy, stratified analyses were conducted for migrant and Finnish-born users of BZDR. The results of unadjusted and adjusted logistic regression analyses are shown in Table IV for long-term use and Table V for polytherapy.

**Table IV.** Unadjusted and adjusted logistic regression analyses, stratified for migrants and Finnish-born participants. Factors associated with long-term use (⩾180 days).

	OR (95% CI)		aOR (95% CI)	
	Migrants	Finnish-born	Migrants	Finnish-born
Sex (ref male)	**1.55 (1.34–1.80)**	**1.68 (1.48–1.80)**	**1.77 (1.49–2.11)**	**1.51 (1.32–1.74)**
Age (ref ⩾60)				
15-29	**0.34 (0.26–0.45)**	**0.47 (0.37–0.60)**	**0.42 (0.31–0.58)**	**0.50 (0.38–0.66)**
30-44	**0.43 (0.34–0.54)**	**0.49 (0.39–0.61)**	**0.56 (0.43–0.74)**	**0.75 (0.58–0.98)**
45-59	**0.55 (0.43–0.69)**	**0.61 (0.48–0.77)**	0.80 (0.61–1.06)	0.97 (0.74–1.26)
SES (ref upper white-collar)				
Lower white-collar	0.80 (0.58–1.11)	1.17 (0.92–1.49)	0.95 (0.67–1.35)	1.26 (0.99–1.61)
Blue collar	**0.70 (0.52**–**0.95)**	**1.70 (1.33**–**2.19)**	0.77 (0.56–1.07)	**1.58 (1.22–2.04)**
Entrepreneurs	0.99 (0.65–1.49)	**2.30 (1.64**–**3.23)**	0.96 (0.62–1.48)	**2.09 (1.49–2.93)**
Others	**1.79 (1.38**–**2.33)**	**4.05 (3.26**–**5.03)**	**1.71 (1.26**–**2.32)**	**3.32 (2.60–4.24)**
Married (ref others)	0.91 (0.78–1.07)	**0.69 (0.61**–**0.79)**	0.94 (0.79–1.12)	**0.82 (0.71–0.96)**
Received social assistance (ref no)	**1.54 (1.32**–**1.79)**	**2.42 (2.09**–**2.79)**	1.10 (0.90–1.34)	**1.60 (1.34–1.92)**
Alcohol-related disorder	**2.42 (1.40**–**4.18)**	**2.67 (1.99**–**3.58)**	1.82 (0.91–3.65)	1.40 (0.999–1.96)
Other substance use disorder	**2.66 (1.36**–**5.17)**	**8.42 (5.84**–**12.15)**	2.02 (0.82–4.95)	**4.49 (2.78–7.23)**
Psychotic disorder	**3.18 (2.26**–**4.46)**	**3.65 (2.77**–**4.81)**	**3.00 (2.02–4.46)**	**2.19 (1.60–2.98)**
Depression	**1.89 (1.45**–**2.46)**	**1.81 (1.45**–**2.26)**	**1.78 (1.30–2.45)**	**1.65 (1.29–2.10)**
Anxiety disorders including PTSD	**2.06 (1.49**–**2.83)**	**1.82 (1.41**–**2.35)**	**1.97 (1.34–2.90)**	**1.67 (1.25–2.22)**
Personality disorder	2.12 (0.99–4.56)	**2.29 (1.51**–**3.48)**	**2.78 (1.19–6.50)**	1.54 (0.95–2.50)

aOR: adjusted for sex, age, SES, social assistance and marital status.

**Table V.** Unadjusted and adjusted logistic regression analyses, stratified for migrants and Finnish-born participants. Factors associated with polytherapy.

	OR (95% CI)		aOR (95% CI)	
	Migrants	Finnish-born	Migrants	Finnish-born
Sex (ref male)	1.00 (0.90–1.10)	**1.12 (1.03**–**1.21)**	1.04 (0.93–1.16)	**1.10 (1.004**–**1.20)**
Age (ref ⩾60)				
15–29	**0.80 (0.66**–**0.97)**	0.93 (0.77–1.12)	**0.76 (0.61**–**0.95)**	0.85 (0.69–1.04)
30–44	0.88 (0.74–1.05)	0.94 (0.79–1.13)	0.86 (0.70–1.04)	0.94 (0.77–1.14)
45–59	0.91 (0.76–1.09)	1.02 (0.85–1.23)	0.89 (0.72–1.09)	1.03 (0.84–1.25)
SES (ref upper white-collar)				
Lower white-collar	1.06 (0.88–1.28)	0.98 (0.87–1.11)	1.08 (0.89–1.32)	0.99 (0.87–1.12)
Blue collar	0.88 (0.74–1.05)	0.95 (0.83–1.09)	0.87 (0.72–1.04)	0.91 (0.79–1.05)
Entrepreneurs	0.86 (0.66–1.10)	1.07 (0.86–1.32)	0.84 (0.64–1.09)	1.02 (0.88–1.17)
Others	0.97 (0.82–1.15)	1.08 (0.96–1.23)	0.94 (0.78–1.13)	1.03 (0.84–1.28)
Married (ref others)	**0.87 (0.78**–**0.96)**	**0.87 (0.80**–**0.95)**	**0.86 (0.77**–**0.96)**	**0.86 (0.79**–**0.95)**
Received social assistance (ref no)	1.10 (0.99–1.22)	**1.22 (1.09**–**1.37)**	1.07 (0.94–1.21)	**1.17 (1.03**–**1.34)**
Alcohol-related disorder	1.57 (0.999–2.46)	1.10 (0.85–1.43)	1.57 (0.94–2.62)	0.94 (0.71–1.25)
Other substance use disorder)	**2.25 (1.31**–**3.87)**	**1.85 (1.28**–**2.66)**	**2.19 (1.16**–**4.11)**	1.33 (0.83–2.12)
Psychotic disorder	1.00 (0.72–1.38)	0.95 (0.73–1.25)	1.07 (0.75–1.52)	0.86 (0.65–1.15)
Depression	1.22 (0.993–1.50)	**1.25 (1.05**–**1.48)**	**1.29 (1.02**–**1.62)**	**1.27 (1.06**–**1.51)**
Anxiety disorders including PTSD	1.11 (0.85–1.45)	1.14 (0.93–1.39)	1.56 (0.86–2.82)	1.15 (0.79–1.67)
Personality disorder	**2.24 (1.26**–**4.01)**	1.18 (0.83–1.68)	1.19 (0.89–1.60)	1.10 (0.88–1.36)

aOR: adjusted for sex, age, SES, social assistance and marital status.

Among both migrant and Finnish-born participants, being female was associated with long-term use of BZDR. Younger users (15–29 and 30–44 years) were less likely to be long-term users than those over 60 years among both migrants and Finnish-born users. Among Finnish-born users, the likelihood of long-term use was higher among blue-collar workers, entrepreneurs and those not in employment when compared with upper white-collar workers. The highest OR was reported among those not in employment (adjusted OR 3.32, 95% CI 2.60–4.24). Among migrants, only those not in employment significantly differed from upper white-collar workers (aOR 1.71, 95% CI 1.26–2.32). Being married was associated with lower likelihood of long-term use among Finnish-born users, but no association was found among migrants. Having a previous diagnosis of other substance use disorder was associated with long-term use of BZDR only among Finnish-born participants. Having personality disorder was associated with long-term use only among migrant participants. Having a psychotic disorder, depression or anxiety disorder were associated with long-term use among both migrants and Finnish-born participants.

Fewer associations were found between background variables and polytherapy. Among migrants, young age (15–29 years) and being married were associated with decreased likelihood of polytherapy. Having a substance use disorder, depression or personality disorder were associated with increased likelihood of polytherapy. Among Finnish-born users, married participants were less likely to use polytherapy. Male participants and those who received social assistance or had depression were more likely to be polytherapy users.

## Discussion

Although it is already known that migrants are less likely to purchase BDZR than Finnish-born people [23], this study shows that if migrants initiate using BDZR, they are less likely to become long-term users or polytherapy users compared to the Finnish-born population. The duration of the first period of BDZR was very similar among Finnish-born participants and different migrant groups.

In our earlier study we found that migrants from Asia and Sub-Saharan Africa are least likely to purchase anxiolytics [23]. In this study focusing on the users, migrants from Sub-Saharan Africa were the most likely to stop the medication once initiated. Migrants from most other regions were also less likely to become long-term users or use several benzodiazepines simultaneously. The factors associated with long-term use were quite similar among both groups, but stronger associations with indicators of low SES and substance use problems were found among Finnish-born users. Only few background variables for the use of polytherapy were identified in both groups.

Since we do not know the needs of the participants or other types of pharmacological and non-pharmacological treatment they receive, it cannot be concluded if migrants with anxiety, sleep problems or other mental problems get less adequate treatment than Finnish-born patients. It is possible that migrants are more often prescribed BZDR only temporarily, as generally recommended and other types of drugs, such as antidepressants, are used if there is a need for long-term treatment. However, our previous study showed that migrants with depression are less likely to initiate and continue using antidepressant medication compared with Finnish-born patients [27], and we assume that the trend might be similar among those with anxiety disorder. It is also known that migrants with depression and anxiety have less visits in specialised psychiatric services when compared with Finnish-born patients [28], which suggests that the needs of migrant patients may not be addressed.

Using BZDR for more than six months or using several drugs concomitantly does not always indicate dependence or abuse [10], but it may be the case for some participants and some of them are likely to use multiple substances. In this study, very few differences between migrants and Finnish-born people were found in the association between psychiatric disorders and use of BZDR, except for substance use disorders. The association between preceding diagnosis of substance use-related disorder, which suggests a severe substance use problem, and long-term use of benzodiazepines was particularly common among Finnish-born participants. It is known that binge drinking [29] and alcohol-related deaths [24] are more common among Finnish-born population than among migrants. It was also found that not being in employment and receiving social assistance were more strongly associated with long-term use of BZDR among Finnish-born participants than migrants.

Attitudes of migrant patients or health professionals may also affect the patterns of using or prescribing BZDR. In Switzerland, migrant patients in a psychiatric hospital had more negative attitudes towards medication compared with non-migrants, and most non-European patients believed that medication effects them differently than local patients [30]. However, more positive attitudes towards psychotropic medication were found among psychiatric patients with migrant background compared with non-migrant patients in Germany [31]. In the United States, less favourable beliefs about psychotropic medication for anxiety have been reported among ethnic minorities [32].

Strengths of this study include the large sample size and detailed information on drug purchases that allows study of the duration of treatment and different patterns of use. There are also limitations. We had no information on the purchases of all drugs for the whole follow-up period and had to exclude those which were not reimbursed. In addition, we could not include drugs that were bought without prescription, which indicates more severe misuse. Even though the sample was large, the statistical power would have been insufficient for more detailed analysis related to specific drugs or diagnoses. The indicator of SES may be less reliable among migrants than among Finnish-born participants. Information on the reason for migration was lacking.

## Conclusion

Migrants use fewer BZDR than Finnish-born population and they are less likely to become long-term users or use several BZDR simultaneously. This is likely to be associated with fewer adverse effects and lower risk of addiction. This type of pattern is generally recommended. However, the results may also indicate poorer access to treatment for anxiety and sleep problems among migrants compared with Finnish-born people.

## Supplemental Material

sj-docx-1-sjp-10.1177_14034948221112470 – Supplemental material for Use of benzodiazepine and related drugs in migrants and Finnish-born persons: a nationwide register-based study Click here for additional data file. Supplemental material, sj-docx-1-sjp-10.1177_14034948221112470 for Use of benzodiazepine and related drugs in migrants and Finnish-born persons: a nationwide register-based study by Venla Lehti, Essi Salama, Solja Niemelä, Antti Tanskanen, Mika Gissler, Jaana Suvisaari and Heidi Taipale in Scandinavian Journal of Public Health
