# Supplementary material for: Use of benzodiazepine and related drugs in migrants and Finnish-born persons: a nationwide register-based study
Source: Scand J Public Health. 2022 Jul 25;51(8):1222–30. doi: 10.1177/14034948221112470 (PMC10642218; doi:10.1177/14034948221112470)
Supplement: sj-docx-1-sjp-10.1177_14034948221112470 – Supplemental material for Use of benzodiazepine and related drugs in migrants and Finnish-born persons: a nationwide register-based study [file sj-docx-1-sjp-10.1177_14034948221112470.docx]

**Supplementary Table 1. Distribution of specific drugs used as a first drug or at any point during follow-up**

|  | **Migrant, % (n)** | **Finnish, % (n)** | **p** |
| --- | --- | --- | --- |
| First drug |  |  |  |
| Oxazepam | 18.4 (1608) | 20.0 (2276) | 0.006 |
| Diazepam | 5.1 (441) | 5.8 (658) | 0.026 |
| Alprazolam | 6.9 (603) | 7.1 (806) | 0.655 |
| Clonazepam | 2.0 (175) | 2.2 (251) | 0.348 |
| Lorazepam | 1.5 (127) | 1.3 (149) | 0.392 |
| Zopiclone | 39.7 (3463) | 34.4 (3917) | <0.001 |
| Zolpidem | 24.9 (2170) | 27.2 (3092) | <0.001 |
| Polytherapy | 1.6 (142) | 2.1 (239) | 0.012 |
|  |  |  |  |
| Drugs used during follow-up |  |  |  |
| Oxazepam | 23.2 (2025) | 25.7 (2927) | <0.001 |
| Diazepam | 6.5 (568) | 7.9 (901) | <0.001 |
| Alprazolam | 8.8 (764) | 9.0 (1021) | 0.617 |
| Clonazepam | 2.8 (244) | 3.3 (373) | 0.053 |
| Lorazepam | 2.1 (184) | 1.9 (218) | 0.335 |
| Zopiclone | 44.0 (3845) | 39.2 (4459) | <0.001 |
| Zolpidem | 28.9 (2524) | 31.0 (3525) | 0.002 |

**Supplementary Table 2. Risk of long-term use and polytherapy by migration status during the first year. Unadjusted and adjusted binary logistic regression analyses. Participants who had died, emigrated from Finland or been hospitalized for more than 90 days during the first year since initiation of BZDR use were excluded.**

|  | **Long-term use (≥180 days)** | | | **Polytherapy** | | |
| --- | --- | --- | --- | --- | --- | --- |
|  | **% (n)** | **OR (95 % CI)** | **aOR (95 % CI)** | **% (n)** | **OR (95 % CI)** | **aOR (95 % CI)** |
| **Migrant status** |  |  |  |  |  |  |
| Finnish-born (ref) | 9.3 (1057) |  |  | 27.8 (3147) |  |  |
| Migrant | 8.8 (766) | 0.94 (0.85-1.04) | **0.78 (0.70-0.89)** | 25.1 (2175) | **0.87 (0.82-0.93)** | **0.90 (0.84-0.97)** |
| **Region of origin** |  |  |  |  |  |  |
| Finland (ref) | 9.3 (1057) |  |  | 27.8 (3147) |  |  |
| EU/EFTA, North America or Australia | 9.4 (266) | 1.01 (0.88-1.17) | 0.93 (0.79-1.10) | 27.0 (759) | 0.96 (0.87-1.05) | 1.00 (0.90-1.11) |
| Russia and Eastern Europe | 9.5 (290) | 1.02 (0.89-1.16) | **0.82 (0.70-0.97)** | 25.8 (789) | **0.90 (0.82-0.99)** | 0.94 (0.85-1.04) |
| North Africa and Middle East | 9.2 (110) | 0.98 (0.80-1.21) | **0.58 (0.45-0.74)** | 21.8 (261) | **0.73 (0.63-0.84)** | **0.69 (0.58-0.81)** |
| Sub-Saharan Africa | 5.4 (29) | **0.56 (0.38-0.81)** | **0.42 (0.27-0.66)** | 23.2 (124) | **0.78 (0.64-0.96)** | 0.81 (0.64-1.03) |
| Asia | 6.3 (53) | **0.66 (0.49-0.87)** | **0.67 (0.49-0.92)** | 21.0 (176) | **0.69 (0.58-0.82)** | **0.74 (0.61-0.89)** |
| **Time since migration** |  |  |  |  |  |  |
| Finnish-born (ref) | 9.3 (1057) |  |  | 27.8 (3147) |  |  |
| Less than 5 years | 8.1 (195) | **0.85 (0.73-0.998)** | **0.73 (0.59-0.90)** | 22.8 (553) | 1.08 (0.98-1.19) | 1.08 (0.97-1.20) |
| 5-15 years | 8.4 (265) | 0.89 (0.77-1.03) | **0.76 (0.64-0.89)** | 25.3 (798) | **0.83 (0.73-0.95)** | 0.86 (0.74-1.01) |
| More than 15 years | 9.5 (235) | 1.02 (0.88-1.18) | **0.83 (0.70-0.98)** | 26.3 (5150) | 0.95 (0.84-1.07) | 0.98 (0.86-1.11) |

aOR=adjusted for sex, age, SES, social assistance, marital status, alcohol use disorder, other substance use disorder, psychosis, depression, anxiety (incl PTSD) and personality disorder
